# Infection with street strain rabies virus induces modulation of the microRNA profile of the mouse brain

**DOI:** 10.1186/1743-422X-9-159

**Published:** 2012-08-11

**Authors:** Pingsen Zhao, Lili Zhao, Kun Zhang, Hao Feng, Hualei Wang, Tiecheng Wang, Tao Xu, Na Feng, Chengyu Wang, Yuwei Gao, Geng Huang, Chuan Qin, Songtao Yang, Xianzhu Xia

**Affiliations:** 1 Institute of Laboratory Animal Sciences, Chinese Academy of Medical Sciences & Peking Union Medical College, Beijing, 100021, China; 2 Key Laboratory of Jilin Province for Zoonosis Prevention and Control, Institute of Military Veterinary, Academy of Military Medical Sciences, Changchun, 130122, China; 3 College of Animal Science and Veterinary Medicine, Jilin University, Changchun, 130062, China; 4 Medical College of Soochow University, Suzhou, 215123, China

**Keywords:** Street strain rabies virus, Brain infection, MicroRNA profiling, Gene profiling, Target prediction, Functional enrichment

## Abstract

**Background:**

Rabies virus (RABV) causes a fatal infection of the central nervous systems (CNS) of warm-blooded animals. Once the clinical symptoms develop, rabies is almost invariably fatal. The mechanism of RABV pathogenesis remains poorly understood. Recent studies have shown that microRNA (miRNA) plays an important role in the pathogenesis of viral infections. Our recent findings have revealed that infection with laboratory-fixed rabies virus strain can induce modulation of the microRNA profile of mouse brains. However, no previous report has evaluated the miRNA expression profile of mouse brains infected with RABV street strain.

**Results:**

The results of microarray analysis show that miRNA expression becomes modulated in the brains of mice infected with street RABV. Quantitative real-time PCR assay of the differentially expressed miRNAs confirmed the results of microarray assay. Functional analysis showed the differentially expressed miRNAs to be involved in many immune-related signaling pathways, such as the Jak-STAT signaling pathway, the MAPK signaling pathway, cytokine-cytokine receptor interactions, and Fc gamma R-mediated phagocytosis. The predicted expression levels of the target genes of these modulated miRNAs were found to be correlated with gene expression as measured by DNA microarray and qRT-PCR.

**Conclusion:**

RABV causes significant changes in the miRNA expression profiles of infected mouse brains. Predicted target genes of the differentially expression miRNAs are associated with host immune response, which may provide important information for investigation of RABV pathogenesis and therapeutic method.

## Background

The rabies virus (RABV), a member of the family *Rhabdoviridae*, is a highly neurotropic virus that can cause fatal infections of the central nervous systems (CNS) of warm-blooded animals [1,2]. Although significant advances have been made in rabies prevention and control, the disease remains a major threat to public health. It causes 55,000 people die around the world every year [3]. Despite the catastrophic clinical outcome of RABV encephalomyelitis, the histopathological changes observed in the CNS are typically relatively mild, showing varying degrees of mononuclear inflammatory cell infiltration of the leptomenings, perivascular cuffing, microglial activation, and neuronophagia. Although there are several hypotheses under active study at present, the pathogenesis of the rabies virus has not yet been determined.

MiRNA (miRNA, miR) is endogenous ≈ 22 nt RNA that negatively regulates gene expression by translational repression [4]. It binds to the complementary sequences in the mRNAs and blocks the translation or accelerates mRNA decay [5]. MiRNAs play key roles in cellular processes such as development, differentiation, cell proliferation, and hematopoiesis [6-9]. Recently, evidence has demonstrated that cellular miRNAs exert regulatory functions in virus-host interactions [10,11]. It is becoming increasingly clear that miRNAs of cellular origin can positively or negatively influence viral infection. For example, miR-122 is indispensable to replication of the hepatitis C virus (HCV), whereas miR-196 and miR-296 substantially attenuate viral replication [12,13]. A recent study reported that miR-28, miR-125b, miR-150, miR-223, and miR-382 inhibit replication of the human immunodeficiency virus (HIV) in CD4^+^ T cells [14].

Microarray analyses have been recently employed to detect changes in host miRNA expression, which can help reveal molecular pathways that govern viral pathogenesis. By using miRNA microarray profiling, researchers have observed differentially expressed patterns of cellular miRNAs in the lungs of mice infected with influenza virus [15]. Another study found miRNAs to be significantly regulated in mouse brains upon Venezuelan equine encephalitis virus (VEEV) infection [16]. Our own recent findings suggest that infection with laboratory-fixed rabies virus, ERA (Evelyn Rokitnicki Abelseth), can induce modulation of the microRNA profile of the mouse brain [17]. However, no report has yet been made regarding the assessment of the host miRNA expression profile in mouse brains upon infection with the street strain of RABV.

In this study, we performed an expression profile of cellular miRNAs in the brains of mice infected with the highly pathogenic street rabies virus. Meawhile,we performed target prediction and functional enrichment of the differentially expressed miRNAs. It was shown that several miRNAs were modulated in mouse brains infected with RABV. Finally, we performed gene microarray analysis and qRT-PCR measurement to verify the expression levels of the predicted targets of the modulated miRNAs in these pathways. The results of functional enrichment revealed that many of the predicted targets of these miRNAs play key roles in the immune response, which are known to be associated with the pathogenesis of RBAV.

## Results

### Characterization of pathogenicity of RABV Fujian strain in mice

**Figure 1:**
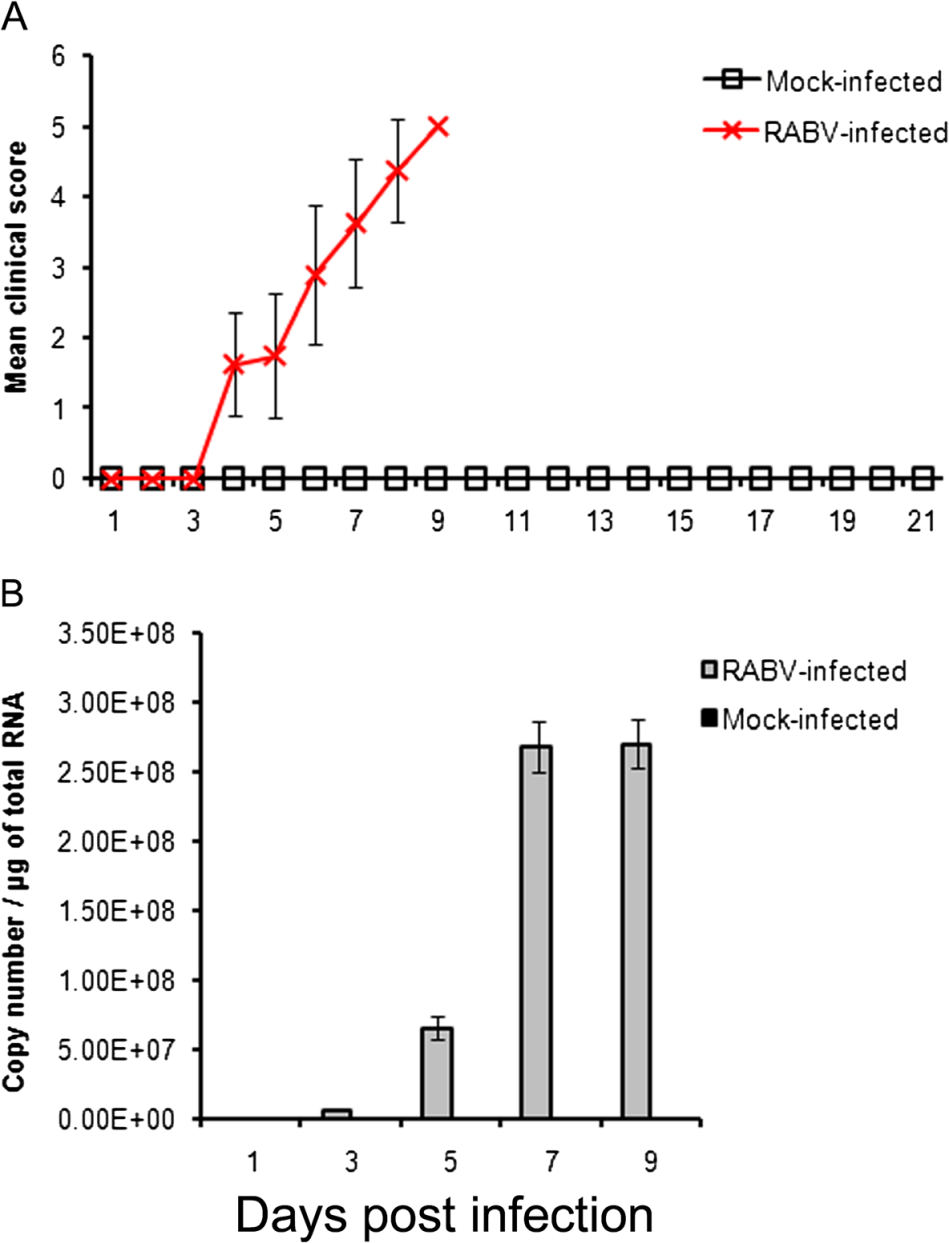
**Outcomes of mice infected with RABV Fujian strain.** After i.c. injection of 10^5^ ffu of street Fujian RABV strain, (**A**) clinical score and (**B**) copy number of RABV N mRNA were recorded as described in Materials and Methods. Mice were monitored for survival for 21 days. Data were obtained from 8 mice (three mice for N mRNA) in each group. Data are the mean ± standard deviation (SD) of one representative experiment. Similar results were obtained in three independent experiments.

All infected animals showed RABV-specific symptoms that increased in severity in a time-dependent manner. After inoculation, all mice developed the clinical signs of disordered movement at 4 days post-infection (dpi). At 6 dpi, considerable aggravation of typical clinical signs was observed, with the onset of trembling, shaking, anger, and hyperexcitation followed by general paralysis (Figure 1A). As presented in a previous study, out of all infected animals, 12.5%, 25%, and 50% were dead at 6, 7, and 8 dpi, respectively, and all mice succumbed to RABV at or before 9 dpi [18]. Viral load in the brain was monitored up to 9 dpi using Taqman qRT-PCR (Figure 1B). RABV replicated rapidly in brain with an increase in copy number from 3 dpi and reached a maximal viral load at 7 dpi. The results demonstrate that the RABV Fujian strain is highly pathogenic in mice.

### Modulation of miRNA profile in brain in response to RABV infection

**Figure 2:**
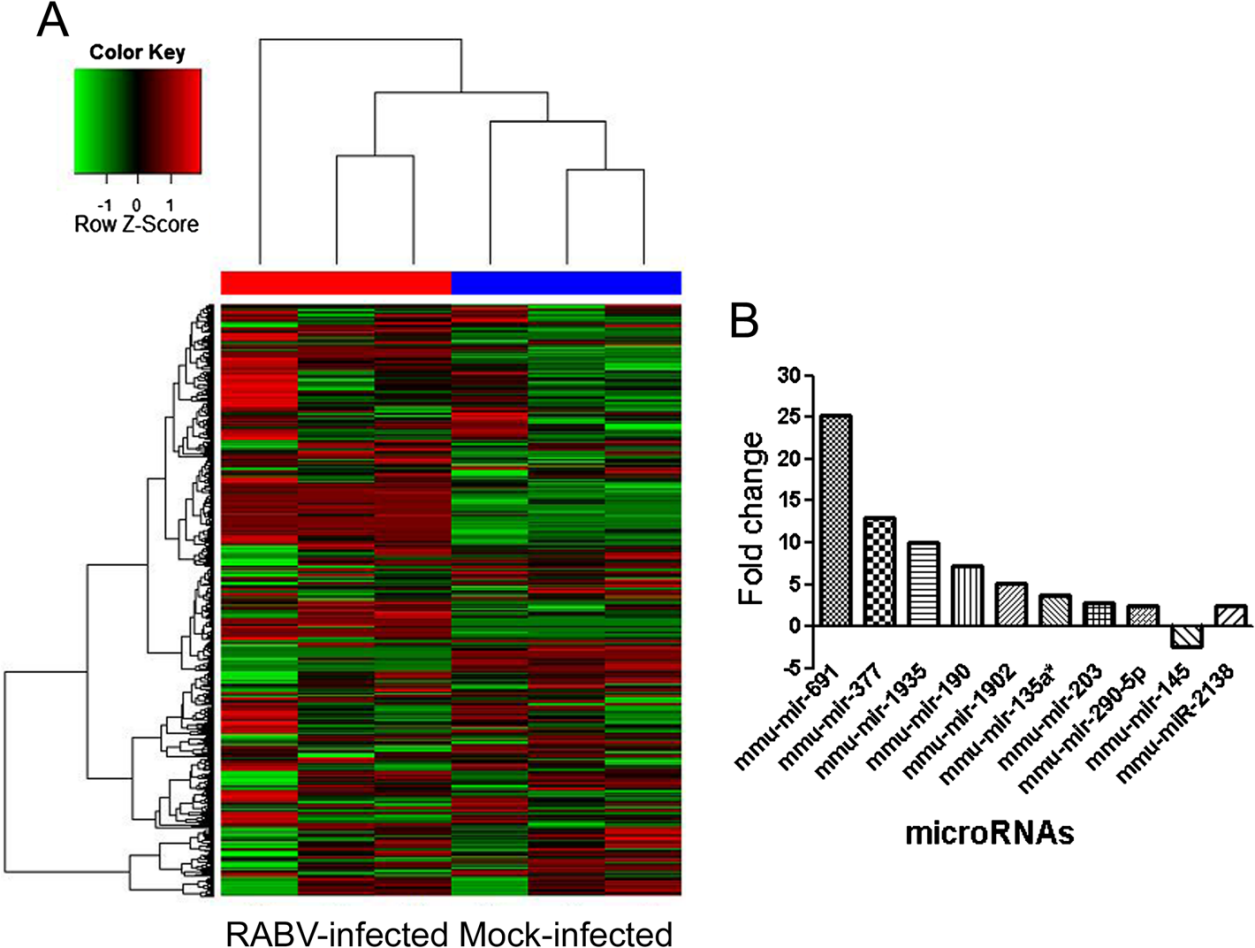
**MiRNA profile of street RABV-infected mouse brain.** (**A**) Two-way hierarchical cluster heat map showing all significantly expressed miRNAs in three independent samples (*P* < 0.01). Each row shows the relative expression level of a single miRNA. Each column shows the expression level of a single sample. Up-regulated miRNAs are shown in red and down-regulated miRNAs are shown in green. Significantly differentially expressed miRNAs in mouse brain upon RABV infection by microarray analysis. MiRNAs whose relative expression levels showed a fold change (FC) ≥ 2 and *P* ≤ 0.01 were considered significantly up-regulated, and those with FC ≤ −2 and *P* ≤ 0.01 were considered significantly down-regulated.

To determine changes in miRNA expression in mouse brains in response to street RABV infection, we evaluated miRNA expression profiles at 7 dpi. The two-way hierarchical cluster heat map clear showed different expression pattern of host miRNAs between RABV and mock infections (Figure 2A). MiRNAs whose relative expression levels showed a fold change (FC) ≥ 2 and *P* ≤ 0.01 were considered significantly up-regulated, and those with FC ≤ −2 and *P* ≤ 0.01 were considered significantly down-regulated. As shown in Figure 2B, nine miRNAs, miR-691, miR-377, miR-1935, miR-190, miR-1902, miR-135a*, miR-203, miR-2138, and miR-290-5p, were found to be significantly up-regulated. However, only one miRNAs, miR-145, was found to be down-regulated upon RABV infection. This indicates that host miRNAs were modulated in the CNS upon infection with street rabies virus.

### Confirmation of differentially expressed miRNAs by qRT-PCR

**Figure 3:**
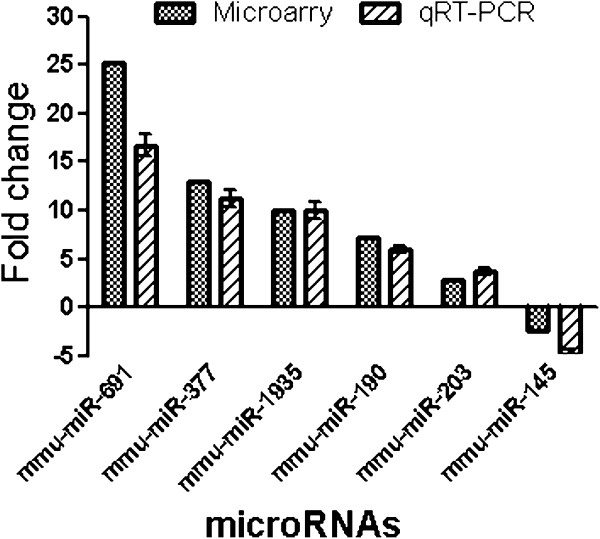
**Verification of differentially expressed miRNAs by qRT-PCR.** Six differentially expressed miRNAs were selected from miRNA microarray datasets and examined by qRT-PCR. The fold change from the qRT-PCR was determined using the 2^-ΔΔCt^ method and all miRNA expression values were normalized against the U6 endogenous control. Data from qRT-PCR are shown as mean ± standard deviation (SD) of one representative experiment. Similar results were obtained in three independent experiments.

To validate the differential expression profiles of miRNAs obtained by microarray analysis, quantitative RT-PCR was performed on six selected differentially expressed miRNAs including miR-691, miR-377, miR-1935, miR-190, miR-203, and miR-145. The data demonstrate that the overall results of qRT-PCR were consistent with those of the microarray analysis. Although differences were observed between these two types of analysis due to intrinsic differences between the techniques, the qRT-PCR results showed the same relative regulation of differentially expressed miRNAs as the microarray data results (Figure 3).

### Target prediction and functional analysis of differentially expressed miRNAs

**Figure 4:**
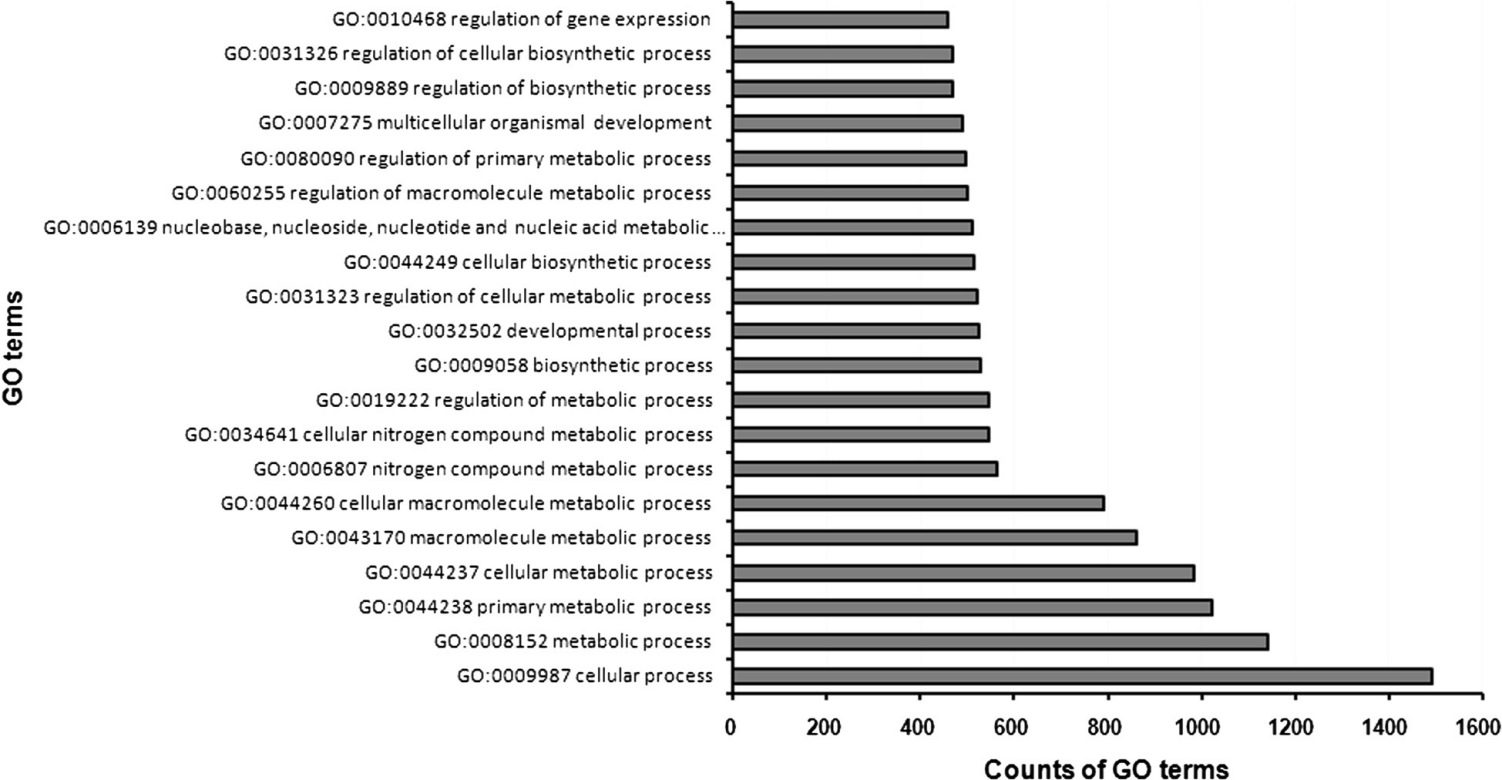
**Enriched GO terms in the biological process category among differentially expressed miRNAs.** After miRNA microarray assay, significantly enriched GO analysis in the biological process category was performed on differentially expressed genes in the brains of RABV-infected mice using DAVID (*P* < 0.01). Only the top twenty GO terms are listed here. For other enriched GO terms, please see Additional file 1: Table S1.

Target genes regulated by these differentially expressed miRNAs were predicted using TargetScan Mouse, MicroCosm, and miRanda. For these ten differentially expressed miRNAs, TargetScan predicted 2,058, MicroCosm predicted 5,433 and miRanda predicted 29,742 target genes. Of these, 3,038 target genes were predicted under all three systems (Additional file 1: Figure S1). Gene ontology (GO) analysis in the Database for Annotation, Visualization and Integrated Discovery (DAVID) was performed for these miRNAs using the predicted gene targets [19]. Functional analysis revealed 106 GO terms to be involved in biological processes, 14 in molecular function, and 20 in cellular components (*P* < 0.01) (Additional file 2: Table S1). The twenty most common GO categories were cellular processes, metabolic processes, cellular metabolic processes, macromolecule metabolic processes, and cellular macromolecule metabolic process (Figure 4). These analyses suggest that cellular miRNAs may regulate cellular metabolic processes during street RABV infection, either directly or indirectly.

### Pathway analysis of target genes of differentially expressed miRNAs

To identify the biological pathways that become active in the mouse brain in response to RABV infection, we mapped the target genes of differentially expressed miRNAs to canonical signaling pathways in the Kyoto Encyclopedia of Genes and Genomes (KEGG). The results showed that 25 statistically remarkable categories (*P* < 0.05) were enriched (Additional file 3: Table S2). As shown in Table 1, the predicted target genes of six up-regulated miRNAs, miR-691, miR-377, miR-1935, miR-190, miR-203, and miR-135a*, and one down-regulated miRNA, miR-145, were found to be involved in immune-related pathways, such as the Jak-STAT signaling pathway, MAPK signaling pathway, Fc gamma R-mediated phagocytosis and cytokine-cytokine receptor interactions. The predicted target genes of five up-regulated miRNAs, miR-691, miR-377, miR-190, miR-203, and miR-1290-5p, and one down-regulated miRNA, miR-145, were found to be involved in other pathways, such as the Adherens junction, Wnt signaling pathway, Axon guidance, cell cycle, TGF-beta signaling pathway, and Focal adhesion.

**Table 1:** Predicted targets of modulated miRNAs upon RABV infection involved in immune response pathways

**miRNA**	**KEGG pathway**	**Target genes**	**Number of targets**	***P*value**
mmu-miR-377	mmu04630: Jak-STAT signaling pathway	*CSF2RB2, EP300, JAK2, SOCS4, BCL2L*	5	0.01
mmu-miR-377	mmu04010: MAPK signaling pathway	*CACNA2D1, FASL, PPM1A, PPP3R1, RASA1, FLNC, PLA2G4A, SRF*	8	0.02
mmu-miR-377	mmu04060: Cytokine-cytokine receptor interaction	*CSF2RB2, FASL, ACVR2A, VEGFA, IL18RAP, KITL, PDGFRA, LTB*	8	0.001
mmu-miR-377	mmu04666: Fc gamma R-mediated phagocytosis	*WASF1, LIMK1, PRKCD, PLA2G4A, RPS6KB1*	5	0.01
mmu-miR-377	mmu04520: Adherens junction	*WASF1, EP300, YES1, PVRL4, PTPRB, SMAD4*	6	1.18E-06
mmu-miR-377	mmu04310: Wnt signaling pathway	*EP300, WNT5A, PPP3R1, FZD3, FZD4, AXIN1, BTRC, SMAD4, CUL1, DKK1*	10	3.62E-04
mmu-miR-377	mmu04360: Axon guidance	*PPP3R1, RASA1, LIMK1, SEMA6A, SEMA3D*	5	0.001
mmu-miR-377	mmu04110: Cell cycle	*EP300, ESPL1, CDC26, CUL1*	4	0.007
mmu-miR-377	mmu04350: TGF-beta signaling pathway	*EP300, ACVR2A, THBS1, PITX2, SMAD4, CUL1, LEFTY2, RPS6KB1*	8	6.96E-06
mmu-miR-377	mmu04510: Focal adhesion	*MYLK2, XIAP, ITGA6, VEGFA, THBS1, PDGFRA, FLNC, LAMC1*	8	0.018
mmu-miR-691	mmu04630: Jak-STAT signaling pathway	*GHR, SPRY2, TYK2, SPRED2, IL12A, CLCF1*	6	0.01
mmu-miR-691	mmu04010: MAPK signaling pathway	*PLA2G10, MKNK2, MAPKAPK3, CACNB2, TGFBR2, DUSP9*	5	0.02
mmu-miR-691	mmu04060: Cytokine-cytokine receptor interaction	*GHR, ACVR2A, BMP2, IL25, AMHR2, IL12A, TGFBR2, TNFSF14, CLCF1*	9	0.001
mmu-miR-691	mmu04666:Fc gamma R-mediated phagocytosis	*LYN, INPP5D*	2	0.01
mmu-miR-691	mmu04520: Adherens junction	*CSNK2B, SRC, CDH1, TGFBR2*	4	1.18E-06
mmu-miR-691	mmu04310: Wnt signaling pathway	*DKK2, CSNK2B, CAMK2G, CTBP2, VANGL1*	5	3.62E-04
mmu-miR-691	mmu04360: Axon guidance	*PTK2, SEMA6D, ABLIM1, NRP1, PLXNA2, SRGAP3, SEMA5A*	7	0.001
mmu-miR-691	mmu04110: Cell cycle	*YWHAB, TFDP1*	2	0.007
mmu-miR-691	mmu04350: TGF-beta signaling pathway	*ACVR2A, BMP2, THBS1, AMHR2, TFDP1, SP1, TGFBR2*	7	6.96E-06
mmu-miR-691	mmu04510: Focal adhesion	*PTK2, IBSP, ITGA10, PGF, CAPN2, THBS1, SRC*	7	0.018
mmu-miR-1935	mmu04630: Jak-STAT signaling pathway	*LIFR, SPRY3*	2	0.01
mmu-miR-1935	mmu04010: MAPK signaling pathway	*MAP3K12, FLNC, MAPT*	3	0.02
mmu-miR-1935	mmu04060: Cytokine-cytokine receptor interaction	*ACVR1, HGF, LIFR, CCR10*	4	0.001
mmu-miR-1935	mmu04666: Fc gamma R-mediated phagocytosis	*MARCKS*	1	0.01
mmu-miR-1935	mmu04520: Adherens junction	*YES1, PVRL1*	2	1.18E-06
mmu-miR-1935	mmu04310: Wnt signaling pathway	*NFAT5*	1	3.62E-04
mmu-miR-1935	mmu04360: Axon guidance	*NFAT5, PLXNA4, SRGAP2, DCC*	4	0.001
mmu-miR-1935	mmu04110: Cell cycle	*STAG1, CDKN1C*	2	0.007
mmu-miR-1935	mmu04510: Focal adhesion	*FLNC*	1	0.018
mmu-miR-190	mmu04630: Jak-STAT signaling pathway	*IL6*	1	0.01
mmu-miR-190	mmu04010: MAPK signaling pathway	*FASL, PLA2G6, BDNF, FGF14*	4	0.02
mmu-miR-190	mmu04060: Cytokine-cytokine receptor interaction	*FASL, HGF, IL6, CXCL16*	4	0.001
mmu-miR-190	mmu04666: Fc gamma R-mediated phagocytosis	*PLA2G6, ARPC5*	2	0.01
mmu-miR-190	mmu04520: Adherens junction	*SMAD2, TJP1*	2	1.18E-06
mmu-miR-190	mmu04310: Wnt signaling pathway	*SMAD2, PPP2R1B, ROCK1, PLCB1*	4	3.62E-04
mmu-miR-190	mmu04360: Axon guidance	*EPHA1, ROCK1*	2	0.001
mmu-miR-190	mmu04110: Cell cycle	*SMAD2, CHEK1, RBL1*	3	0.007
mmu-miR-190	mmu04350: TGF-beta signaling pathway	*SMAD2, PPP2R1B, PITX2, ROCK1, RBL1*	5	6.96E-06
mmu-miR-190	mmu04510: Focal adhesion	*CAPN2, HGF, ROCK1*	3	0.018
mmu-miR-135a*	mmu04630: Jak-STAT signaling pathway	*EP300,IL24,IFNAR2,IL12RB1,IFNG*	5	0.01
mmu-miR-135a*	mmu04060: Cytokine-cytokine receptor interaction	*IL24,IFNAR2,IL12RB1,IFNG,CXCL16,CD40LG*	6	0.001
mmu-miR-135a*	mmu04666: Fc gamma R-mediated phagocytosis	*NCF1*	1	0.01
mmu-miR-135a*	mmu04520: Adherens junction	*EP300, ACTN2*	2	1.18E-06
mmu-miR-135a*	mmu04310: Wnt signaling pathway	*EP300, WNT1, PPP2R1A*	3	3.62E-04
mmu-miR-135a*	mmu04110: Cell cycle	*EP300, YWHAQ, ANAPC7*	3	0.007
mmu-miR-135a*	mmu04350: TGF-beta signaling pathway	*EP300, BMP4, IFNG, PPP2R1A*	4	6.96E-06
mmu-miR-135a*	mmu04510: Focal adhesion	*PTEN, ITGA8, ACTN2*	3	0.018
mmu-miR-290-5p	mmu04630: Jak-STAT signaling pathway	*IL13RA1, IFNGR2, AKT1, CSF2RB*	4	0.01
mmu-miR-290-5p	mmu04010: MAPK signaling pathway	*GADD45A, MAPKAPK3, MEF2C, MAP3K1, AKT1FGF23*	5	0.02
mmu-miR-290-5p	mmu04060: Cytokine-cytokine receptor interaction	*IL13RA1, IL18R1, CD40, IFNGR2, CXCL12, ACVR1B, CSF2RB*	7	0.001
mmu-miR-290-5p	mmu04666: Fc gamma R-mediated phagocytosis	*MARCKS, AKT1*	2	0.01
mmu-miR-290-5p	mmu04520: Adherens junction	*LMO7, ERBB2, PTPRB, LEF1, INSR, SSX2IP, PVRL3*	7	1.18E-06
mmu-miR-290-5p	mmu04310: Wnt signaling pathway	*LEF1, CAMK2G, PLCB1, AXIN2*	4	3.62E-04
mmu-miR-290-5p	mmu04360: Axon guidance	*ROBO1, L1CAM, SEMA5B, CXCL12*	4	0.001
mmu-miR-290-5p	mmu04110: Cell cycle	*SMC3, MCM5, GADD45A, CDC25A, TFDP1, HDAC2, RAD21, ORC5L*	8	0.007
mmu-miR-290-5p	mmu04350: TGF-beta signaling pathway	*TFDP1*	1	6.96E-06
mmu-miR-290-5p	mmu04510: Focal adhesion	*XIAP, ERBB2, AKT1*	3	0.018
mmu-miR-203	mmu04630: Jak-STAT signaling pathway	*IL24, CNTFR, IL22RA2, CCND1, SOCS3, AKT2, IL12B*	7	0.01
mmu-miR-203	mmu04010: MAPK signaling pathway	*CACNA2D1, MAP3K13, DUSP5, MAP4K3, STK3, NLK, AKT2, ATF2, PDGFRA, RAP1A, MAPT, MAP3K1, PPM1B, FGF7, CRK, PRKCA*	16	0.02
mmu-miR-203	mmu04060: Cytokine-cytokine receptor interaction	*MET, IL24, CNTFR, ACVR2A, IL22RA2, VEGFA, XCL1, PDGFRA, IL12B*	9	0.001
mmu-miR-203	mmu04666: Fc gamma R-mediated phagocytosis	*PLD2, AKT2, DNM1L, PPAP2B, CRK, VAV3, PRKCA*	7	0.01
mmu-miR-203	mmu04520: Adherens junction	*MET, NLK, SRC, PTPRB, SNAI2*	5	1.18E-06
mmu-miR-203	mmu04310: Wnt signaling pathway	*SFRP2, CCND1, NLK, APC, CSNK1A1, CUL1, PRKCA*	7	3.62E-04
mmu-miR-203	mmu04360: Axon guidance	*MET, ABL1, SEMA3A, SEMA6A, SEMA5A*	5	0.001
mmu-miR-203	mmu04110: Cell cycle	*YWHAQ, CCND1, ABL1, SKP2, CUL1*	5	0.007
mmu-miR-203	mmu04350: TGF-beta signaling pathway	*ACVR2A, ID2, BMP5, CUL1, ID4*	5	6.96E-06
mmu-miR-203	mmu04510: Focal adhesion	*MET, PXN, CCND1, VEGFA, PPP1C, SRC, PDGFRA, RAP1A, RAPGEF1, TNC, COL4A4, CAV1, CRK, VAV3, PRKCA*	15	0.018
mmu-miR-145	mmu04630: Jak-STAT signaling pathway	*MYC, STAM, STAT4, SOCS7*	4	0.01
mmu-miR-145	mmu04010: MAPK signaling pathway	*MAP3K3, HSPA1L, PPP3CA, DUSP4, MYC, RASA2, RAPGEF2, FLNB, DUSP6, TGFBR2, MAP4K4, CRKL*	12	0.02
mmu-miR-145	mmu04060: Cytokine-cytokine receptor interaction	*FLT3L, TNFRSF11B, IL17RB, INHBB, ACVR1B, TGFBR2*	6	0.001
mmu-miR-145	mmu04666:Fc gamma R-mediated phagocytosis	*ARF6, CFL2, LAT, ARPC5, CRKL*	5	0.01
mmu-miR-145	mmu04520: Adherens junction	*YES1, ERBB2, CTNND1, ACTG1, SMAD3, ACTB, TGFBR2*	7	1.18E-06
mmu-miR-145	mmu04310: Wnt signaling pathway	*PPP3CA, FZD9, MYC, CTNNBIP1, SMAD3, SENP2, WNT5B*	7	3.62E-04
mmu-miR-145	mmu04360: Axon guidance	*PPP3CA, CFL2, SRGAP1, DPYSL2, SEMA3, EFNB3, SRGAP2, ABLIM2, SEMA6A, PLXNA2, SEMA3D*	11	0.001
mmu-miR-145	mmu04110: Cell cycle	*MCM5, MYC, SMAD3, CDK6, SFN, ORC2L*	6	0.007
mmu-miR-145	mmu04350: TGF-beta signaling pathway	*MYC, SMAD3, INHBB, TGFBR2, SMAD5*	5	6.96E-06
mmu-miR-145	mmu04510: Focal adhesion	*ERBB2, ACTG1, FLNB, ACTB*	4	0.018

### DNA microarray assay and qRT-PCR measurement of miRNA targets

MiRNAs predominately function as repressors of target gene expression. The miRNAs and their targets show mutually antagonistic expression levels. To determine whether any such correlation exists between deregulated miRNA levels and their corresponding targets, we performed DNA microarray assay and qRT-PCR validation. To identify the genes involved in the pathways common to miRNA target prediction and RABV infection, we measured the expression of genes from Jak-STAT signaling pathway (*SOCS4*), cytokine-cytokine receptor interactions (*IL25*, *CD40*, *VEGFA*, and *CCR10*), MAPK signaling pathway (*MAPKAPK3*), Fc gamma R-mediated phagocytosis (*ARF*6), and Wnt signaling pathway (*NFAT5*). As presented in Table 2, *MAPKAPK3* and *IL25*, the targets of up-regulated miRNA miR-691; *SOCS4*, the target of up-regulated miRNAs miR-377; *CCR10* and *NFAT5*, the targets of up-regulated miRNA miR-1935; *VEGFA*, the target of up-regulated miRNA miR-203; and *CD40*, the target of up-regulated miRNA miR-290-5p, were found to be down-regulated. *ARF*6, the target of down-regulated miRNA miR-145, was found to be up-regulated. The negative correlation between these miRNAs and their targets showed these pathways to be part of the RABV infection response at the expression level. Together, these results strongly suggest that certain miRNAs may be associated with RABV infection and pathogenesis.

**Table 2:** DNA microarray and qRT-PCR analysis of expression of miRNA targets

**MicroRNA**	**Accession number**	**MiRNA microarray (Fold: ↑ or ↓)**	**qRT-PCR** **(Fold: ↑ or ↓)**	**Targets of microRNAs**	**Accession Number**	**DNA microarray (Fold: ↑ or ↓)**	**qRT-PCR (Fold: ↑ or ↓)**
mmu-miR-691	MI0004659	25.29×,↑	16.71 ± 3.24×,↑	*MAPKAPK3*	NM_178907	1.57×,↓	10.02 ± 4.11×,↓
mmu-miR-691	MI0004659	25.29×,↑	16.71 ± 3.24×,↑	*IL25*	NM_080729	1.45×,↓	12.75 ± 2.91×,↓
mmu-miR-377	MI0000794	12.90×,↑	11.22 ± 2.87×,↑	*SOCS4*	NM_080843	1.35×,↓	9.52 ± 2.56×,↓
mmu-miR-1935	MI0009924	10.06×,↑	9.94 ± 2.63×,↑	*CCR10*	NM_007721	1.39×,↓	4.58 ± 2.03×,↓
mmu-miR-1935	MI0009924	10.06×,↑	9.94 ± 2.63×,↑	*NFAT5*	NM_133957	1.59×,↓	7.69 ± 3.17×,↓
mmu-miR-203	MI0000246	2.79×,↑	3.73 ± 1.42×,↑	*VEGFA*	NM_001025257	1.37×,↓	4.87 ± 1.96×,↓
mmu-miR-290-5p	MI0000388	2.44×,↑	6.79 ± 2.31×,↑	*CD40*	NM_011611	1.3×,↓	5.43 ± 2.76×,↓
mmu-miR-145	MI0000169	0.40×,↓	4.82 ± 1.58×,↓	*ARF6*	NM_007481	1.21×,↑	5.38 ± 2.77×,↑

Data from qRT-PCR are shown as mean ± standard deviation (SD) of one representative experiment. Similar results were obtained in three independent experiments.

## Discussion

RABV, a pathogen well-adapted to the nervous system, infects the neurons of warm-blooded animals. Despite the catastrophic clinical outcome of RABV encephalomyelitis, especially that caused by street viruses, the histopathological changes observed in the CNS are typically relatively mild. CNS has more intrinsic mechanisms for controlling immune response than other organs. Indeed, many viral infections can be cleared from the CNS by immune mechanisms [20]. This may indicate that, upon viral infection, the immune privilege of the CNS is not as strong as has been proposed [21].

In mammals, approximately 30% of all protein-coding genes are predicted to be regulated by miRNAs [22]. Currently, nearly a thousand miRNAs have been cloned, each potentially regulating hundreds of genes by complementary binding to the 3′-untranslated region (3′-UTR) of the target mRNAs. Recent publications have provided compelling evidence that a range of miRNAs are involved in the regulation of immunity, including the development and differentiation of B and T cells, proliferation of monocytes and neutrophils, antibody switching and the release of inflammatory mediators [4,23-25]. More importantly, researchers have reported that cellular miRNAs play key regulatory roles during viral infection and that altered cellular miRNA expression in response to viral infection may be an important determinant of virulence [10,11,26].

Recently, our findings suggested that infection with laboratory-fixed rabies virus strain, ERA, induced modulation of microRNA profile of mouse brains [17]. In the current study, a comprehensive examination of miRNA expression from brains of street-RABV- and mock-infected mice was performed. The results showed that host miRNAs were modulated upon RABV infection. We found that the expression profiles of host miRNAs in mouse brains infected by these two strains of RABVs were completely different from each other. Interestingly, the same differences in transcriptome were observed between the mouse brains upon infection with the two strains of RABVs, ERA and street RABV Fujian strain (unpublished data) [18]. This discrepancy can be partly attributed to differences in virulence of the viruses. Further study is required to determine the correlation between specific miRNA expression and RABV virulence using reverse genetics or RNA interference. Some studies have also used profiling technology to evaluate the modulations in miRNA expression that occur in response to viral infection. For example, Li and colleagues performed miRNA profiling in the lungs of mice infected with influenza and found that cellular miRNA might be a contributing factor to the extreme virulence of the influenza virus [15]. Bhomia and colleagues suggested that host miRNAs were significantly modulated in mouse brains upon VEEV infection [16].

Although many computational approaches have been developed to predict miRNA targets using sequence information, their accuracy is limited [27-30]. To increase reliability, we used three web-based target prediction databases, TargetScan, MicroCosm, and Targets. The pathways listed in Table 1 and Additional file 3: Table S2 are among the most significant, as indicated by predictions from all three algorithms. It has been shown that miRNA-induced down-regulation of target genes provides opportunities to develop new approaches to target identification and validation using high-throughput expression profiling [31]. Gene expression profiling data have been used to identify functional targets of miRNAs [32-34]. MiRNAs and the mRNAs that they target for degradation can be expected to exhibit an inverse expression relationship. Researchers established a strategy for miRNA target identification using these inverse relationships as predicted from the paired expression profiles [35]. In our recently published work, we also identified miRNA targets using these inverse relationships as predicted from the paired expression profiles [17]. In the present study, we simultaneously collected miRNA and DNA microarray data from the same samples and compared the predicted targets of significantly modulated miRNAs to the gene expression profiles of RABV-infected mouse brains. This showed that some of the predicted miRNA targets were correlated with the mRNA expression profile.

Host defense against viral invasion requires induction of appropriate innate immune responses. Upon recognition of viral components, host cells become activated and produce type I IFN and proinflammatory cytokines [36,37]. A suitable amount of type I interferon (IFN) induces cellular resistance to viral infection and apoptosis of virus-infected cells [38]. However, viruses have developed several strategies to evade and subvert the immune responses mediated by type I IFN, including harnessing host miRNAs. A recent study demonstrated that the vesicular stomatitis virus (VSV), family *Rhabdoviridae*, can induce up-regulation of miR-146a, which feedback-inhibits RIG-I-dependent IFN-I production in macrophages [39]. In our study, enrichment of KEGG pathways revealed that the predicted target genes of differentially expressed miRNAs upon RABV infection may involve Jak-STAT signaling pathway. The Jak-STAT pathway is initiated in response to cytokines, such as interleukins and IFNs, and growth factors. To invasive innate immune response of host, RABV interrupts IFN Jak-STAT signaling in a manner of activation-dependent targeting of *STAT1* and *STAT2*[40]. In this study, the target genes of modulated miRNAs were found to be involved in Jak-STAT signaling, including *JAK2SOCS4*, which are targets of miR-377; *TYK2* and *IL12A*, targets of miR-691; *IL6*, a target of miR-190; *IFNAR2*, a target of miR-135a*; *IFNGR2* and *AKT1*, targets of miR-290-5p; and *SOCS3* and *AKT2*, targets of miR-203. This suggests that the Jak-STAT pathway may be affected by RABV-inducible cellular miRNAs (Table 1).

Recent studies have revealed the important regulatory roles played by cytokines and their receptors in RABV infection. One study showed that over-expression of cytokine CCL3 (MIP-1α) in mouse brains decreased RABV pathogenicity [41]. The same research team also demonstrated that MIP-1α not only reduces viral pathogenicity but also enhances immunogenicity by recruiting dendritic cells and B cells to the sites of immunization, lymph nodes, and blood [42]. We observed that several targets of differentially expressed miRNAs are involved in cytokine-cytokine receptor interaction. These included *FASLIL18RAP*, and *KITL*, which are targets of miR-377; *IL25IL12ATNFSF14*, and *CLCF1*, which are targets of miR-691; *CCR10*, a target of miR-1935; *CXCL16*, a target of miR-190; *IL24IFNGCXCL16*, and *CD40LG*, targets of miR-135a*; *IL18R1CD40CXCL12*, and *CSF*, targets of miR-290-5p; and *IL24XCL1*, and *IL12B*, targets of miR-203 (Table 1). Our findings showed that modulated miRNAs may regulate the functions of cytokines during RABV infection.

The MAPK signaling pathway has been shown to regulate the expression of genes involved in the immune response to pathogens. Viral infection can induce activation of the MAPK signaling pathway [43,44]. RABV infection induces MAPK and NF-κB activation, which have been found to regulate chemokine expression in microglial cells [45]. Some key MAPK signaling pathway-related target genes were identified in the present study. These included *SPRED2*, a target of miR-691, *MAP3K12*, a target of miR-1935, *MAPKAPK3* and *MAP3K1*, targets of miR-290-5p, and *MAP3K13MAP4K3*, and *MAP3K1*, targets of miR-203 (Table 1). This demonstrated that RABV-induced cellular miRNAs might be involved in the MAPK signaling pathway after RABV infection.

In summary, the results of the present study provide evidence that specific miRNAs are modulated in the street-RABV-infected brain. This result was found to be completely different from the expression profiles of host miRNAs in the CNS of mice infected with the laboratory-fixed strains of RABV. Considering that this was verified by both DNA microarray and qRT-PCR, we suggest that the modulated miRNAs might affect the biological processes of cells during RABV infection. Our study suggests that host miRNAs might be an important class of targets and may play a key role in regulating gene expression in response to highly pathogenic RABV infection of the CNS.

## Conclusion

In summary, our findings suggested that street RABV infection resulted in significant changes in the expression of multiple miRNAs in mouse brains. The modulated miRNAs might regulate biological processes of cells during RABV infection. The predicted target genes of these differentially expressed miRNAs are involved in immune responses in the host. MiRNA and mRNA profiles obtained in this study might help elucidate the regulatory mechanisms that mediate the host response to RABV exposure.

## Methods

### Viruses

RABV street rabies virus Fujian strain, isolated from a rabid dog in Fujian Province, was used for this study. Viral stocks were prepared as described elsewhere with minor modifications [46]. Briefly, Three-day-old suckling mice were intracerebrally (i.c.) infected with 30 μl of viral sample. When moribund, mice were euthanized and brains were removed. A 10% (wt/vol) suspension was prepared by homogenizing the brain in Dulbecco’s modified Eagle’s medium (DMEM, Gibco CA, U.S.). The homogenate was centrifuged to remove debris, and the supernatant was collected and stored at −80°C. The viral titers were determined in triplicate on monolayer cultures of mouse neuroblastoma cell (NA) as described previously [47].

### Animal infection and assessment of clinical signs

Six-to-eight-week-old female BALB/c mice were obtained from the Changchun Institute of Biological Products, China. Animals had access to food and water ad libitum. All the experiments with live virus challenge were carried out at the bio-safety level 2 (BSL-2) facilities of the Key Laboratory of Jilin Province for Zoonosis Prevention and Control, Institute of Military Veterinary, Academy of Military Medical Sciences.

The animal experiments were conducted with prior approval from the Animal Welfare and Ethics Committee of Institute of Military Veterinary, Academy of Military Medical Sciences under the permit number (SCXK-2002-018). All manipulation of the mice satisfied the requirements of the Regulations of Experimental Animal Administration of China.

Mice in the experimental mice were infected with 10^5^ focus-forming units (FFU) of RABV in 30 μl of DMEM via the i.c. route. Mice in the control group were mock-infected i.c. with DMEM containing uninfected brain homogenates for use as controls. Infected animals were observed twice daily for 21 days for the development of rabies. Disease progression and mortality were monitored. Clinical signs were scored as described elsewhere using a scale of 0 to 5: 0, no clinical signs; 1, disordered movement; 2, ruffled fur, hunched back; 3, trembling and shaking; 4, complete loss of motion (complete paralysis); 5, death [48].

### Tissue collection and total RNA isolation

Tissue collection and total RNA isolation were performed as described elsewhere [18]. Briefly, mice at 7 dpi were anesthetized with ketamine-xylazine (1.98 and 0.198 mg per mouse, respectively) and euthanized. Brains were harvested and stored in RNAlater (Ambion TX, U.S.) at −80°C for total RNA extraction. Total RNAs were isolated from the entire homogenized brains using the mirVana^TM^ miRNA Isolation Kit (Ambion TX, U.S.). The integrity of total RNA was analyzed by Agilent 2100 Bioanalyzer (Agilent Technologies, CA, U.S.).

### Taqman PCR quantification of viral loads

To determine viral load in infected brain tissues, TaqMan qRT-PCR was performed on RNA samples using Custom TaqMan® Gene Expression Assays (Applied Biosystems, CA, U.S.). The primers specific to the N gene of the RABV Fujian strain (forward: 5′-GTGGGTACTGTTGTCACTGCTTA-3′ and reverse: 5′-GTGAGATTTATCTGCTTTATGAACCCTGTA-3′) and probe (FAM-TCCTGAGCAATCTTC-NFQ) and the protocols for TaqMan qRT-PCR were used as described by our previous study [18]. The TaqMan PCR was performed using Brilliant II qPCR Master Mix (Agilent Technologies, CA, U.S.) in an Mx3005P apparatus (Agilent Technologies, CA, U.S.) according to the manufacturer’s instructions. TaqMan runs of experimental samples contained at least three replicates with no-template or no-primer controls. Real-time PCR was performed in reaction mixtures including 12.5 μl of 2× QPCR master mix (Agilent Technologies, CA, U.S.), 1 μl of 20× Custom TaqMan® Gene Expression Assays (Applied Biosystems, CA, U.S.), 0.375 μl of diluted reference dye (Agilent Technologies, CA, U.S.), and nuclease-free PCR-grade water to a final volume of 25 μl. The PCR conditions were (i) 95°C for 2 minutes and (ii) 40 cycles of 95°C for 5 seconds and 60°C for 20 seconds. A standard curve was generated from serially diluted RABV N RNAs of known copy numbers, and the copy numbers of samples were normalized to 1 μg of total RNA. An absolute standard curve method was to calculate the copy numbers of RABV N mRNA in mouse brain tissue [49]. To exclude contamination of genomic DNA, control cDNA reactions in which reverse transcriptase was omitted were prepared in parallel as described elsewhere [50]. These were uniformly negative.

### μParaflo miRNA microarray assays

Three RABV-infected and mock-infected mice at 7 dpi were randomly selected for miRNA microarray analysis. μParaflo miRNA microarray assays were outsourced to LC Sciences (Houston, TX, U.S.). The assay was performed on a 5 μg total RNA sample, which was size-fractionated using a YM-100 Microcon centrifugal filter (Millipore, MA, U.S.). The small RNAs (<300 nt) isolated were 3′-extended with poly-(A) tails using poly-(A) polymerases. An oligonucleotide tag was then ligated to each poly-(A) tail for later fluorescent dye staining. Hybridization was performed overnight on a μParaflo microfluidic chip using a micro-circulation pump (Atactic Technologies, TX, U.S.) [51,52]. The microfluidic chips each contained a detection probe consisting of a chemically modified nucleotide coding segment complementary to target microRNA (from miRBase, http://microrna.sanger.ac.uk/sequences/) or other RNA (control or customer defined sequences) and a spacer segment of polyethylene glycol to extend the coding segment away from the substrate. The detection probes were made by in situ synthesis using PGR (photogenerated reagent) chemistry. The hybridization melting temperatures were balanced by chemical modifications of the detection probes. Hybridization used 100 μl 6xSSPE buffer (0.90 M NaCl, 60 mM Na_2_HPO_4_, 6 mM EDTA, pH 6.8) containing 25% formamide at 34°C. Post-hybridization detection used fluorescence labeling with tag-specific Cy5 dyes. Hybridization images were collected using a laser scanner GenePix 4000B (Molecular Devices) and digitized using Array-Pro image analysis software (Media Cybernetics, MD, U.S.). Raw data were obtained for further analysis.

### DNA microarray assays

Three mice at 7 dpi were randomly selected from the RABV- and mock-infected groups for DNA microarray analysis. mRNA microarray assays were outsourced to Phalanx Biotech Group. Inc. (Hsinchu, Taiwan). Fluorescence-labeled cRNA was prepared from 5 μg of total RNA using a Message AMPTM aRNA Kit (Ambion, TX, U.S.) and Cy5 dye (Amersham Pharmacia, NJ, U.S.). Fluorescent targets were hybridized to the Mouse OneArray^TM^ Whole Genome DNA microarray (Phalanx, Hsinchu, Taiwan) containing 31,802 oligonucleotides, including 29,922 mouse genome probes, and 1,880 experimental control probes. After an overnight hybridization at 50°C, non-specific binding targets were washed in three different washing steps, and the slides were dried by centrifugation and scanned using an GenePix 4000B (Molecular Devices, CA, U.S.). The Cy5 fluorescent intensity of each spot was analyzed using GenePix 4.1 (Molecular Devices, CA, U.S.). Raw data were obtained for further analysis.

### Quantitative real-time PCR

The protocols for qRT-PCR of miRNAs and mRNAs were used as described in our previous study [17]. The expression of specific miRNAs was analyzed with quantitative real-time PCR using a LNA^TM^-based qRT-PCR kit, miRCURY LNA^TM^ Universal RT microRNA PCR System (Exiqon, Denmark) according to manufacturer’s instructions. Total RNA from brain tissues of RABV-infected and mock-infected mice was converted to cDNA using universal reverse transcriptase primers (Exiqon, Denmark). cDNA samples were diluted 1:80 in nuclease-free water and then PCR was amplified using SYBR Green Master Mix and specific LNA^TM^ miRNA primers (Exiqon, Denmark) for mmu-miR-691 (target sequence AUUCCUGAAGAGAGGCAGAAAA), mmu-miR-377 (target sequence AUCACACAAAGGCAACUUUUGU), mmu-miR-1935 (target sequence AGGCAGAGGCUGGCGGAUCUCU), mmu-miR-190 (target sequence UGAUAUGUUUGAUAUAUUAGGU), mmu-miR-203 (target sequence GUGAAAUGUUUAGGACCACUAG), and mmu-miR-145 (target sequence GUCCAGUUUUCCCAGGAAUCCCU). Samples were run in duplicate on an Mx3005P QPCR system (Agilent Technologies, CA, U.S.). The data were normalized to a U6 RNA control (Exiqon, Denmark) and relative expression was calculated using the 2^-ΔΔCt^ method [53].

For mRNA quantitative real-time PCR, reverse transcription was performed using a Reverse Transcription System (Promega, WI, U.S.) according to manufacturer’s instructions. The names of the genes and their primers are given in Table 3. Real-time PCR was performed using Brilliant® II SYBR® Green QPCR Master Mix (Agilent Technologies, CA, U.S.) in an Mx3005P QPCR system (Agilent Technologies, CA, U.S.) according to the manufacturer’s instructions. Expression of the gene of interest was normalized to glyceraldehyde-3-phosphate dehydrogenase (*GAPDH*). The expression levels of genes were measured in terms of threshold cycle value (CT) using the 2^-ΔΔCt^ method [53].

**Table 3:** Primers for selected genes analyzed using quantitative real-time PCR

**Gene symbol**	**Gene title**	**Forward primer 5′ → 3′**	**Reverse primer 5′ → 3′**	**Amplicon (bp)**
*MAPKAPK3*	Mitogen-activated protein kinase-activated protein kinase 3	TATTATGTGGCTCCTGAGGTCCT	TCATCTTCCCACCCTTAGGT	112
*IL25*	Interleukin 25	TGCTTGGAGCGCAGGCTCTA	AGAGAGGGTTGGCCCGTA	113
*SOCS4*	Suppressor of cytokine signaling 4	ATGTTGAAATTCCTCTAAGAAG	CGTAGAACGTCAAAGACAAATT	124
*CCR10*	Chemokine (C-C motif) receptor 10	ACCCAGTGTCTCCCTGATG	TTCCAGTCGGTCCCGGTTGA	136
*NFAT5*	Nuclear factor of activated T-cells 5	CACCTTCTTCCCCCATTTTAT	GTCTTTCAAAAAGGGTTAA	122
*VEGFA*	Vascular endothelial growth factor A	CCTATTCCCCTCTTAAATCGT	AGGAACTGAAGAGAGACCT	125
*CD40*	CD40 antigen	TCGGCTTCTTCTCCAATCAGT	TGAAATTTGGTGTCTACTGT	126
*ARF6*	ADP-ribosylation factor 6	GGAAGGTGCTATCCAAGATCTT	TAACCGGGTCGAAGTTGAACAT	108
*GAPDH*	Glyceraldehyde-3-phosphate dehydrogenase	CTCAACTACATGGTCTACATGTTC	ATTTGATGTTAGTGGGGTCTCGCTC	142

### Microarray data analysis

Data from microarrays were analyzed as described in our previous study [17]. First, raw data from both miRNA and DNA microarrays, with replicates, were averaged. Probes with median detection *P* value > 0.05 and flags reported as absent were filtered out. Expression data were normalized using a variance stabilizing method VSN in a Bioconductor ( http://www.bioconductor.org) [54]. Differential expression of miRNAs were assessed by eBayes (Empirical Bayes Statistics) from LIMMA [55]. Differentially expressed miRNAs with *P* < 0.01 were selected for further analysis. Microarray data of miRNA and target genes were found to be MIAME compliant and have been submitted to the Gene Expression Omnibus (GEO) database ( http://www.ncbi.nlm.nih.gov/geo/; accession number: GSE26269 (miRNA data); GSE26270 (mRNA data)).

### Target prediction and functional enrichment of differentially expressed miRNAs

Target prediction and functional enrichment of differentially expressed miRNAs were completed as described in our previous study [17]. Three continuously updated miRNA target prediction databases, TargetScan, MicroCosm Targets, and miRanda, were used to infer the targets of differentially expressed miRNAs. The intersection of these datasets was taken as reliable. Gene ontology (GO) category and *Kyoto Encyclopedia of Genes and Genomes* (KEGG) pathway analyses of target genes of differentially expressed miRNAs were performed using the web-based tool, Database for Annotation, Visualization, and Integrated Discovery (DAVID, http://david.abcc.ncifcrf.gov/)^19^.

## Abbreviations

RABV, Rabies virus; ERA, Evelyn Rokitnicki Abelseth; CNS, Central nervous system; miRNA, microRNA; qRT-PCR, quantitative reverse transcription polymerase chain reaction; FFU, Focus-forming units; FC, Fold change; GO, Gene Ontology; KEGG, *Kyoto Encyclopedia of Genes and Genomes*; DAVID, Database for Annotation, Visualization and Integrated Discovery; SD, Standard deviation.

## Competing interests

The authors declare that they have no competing interests.

## Authors’ contributions

PSZ conceived of the study, participated in its design and coordination, drafted the manuscript, and performed the animal experiments, sample preparation, qRT-PCR, and data analysis. LLZ assisted with the propagation of virus and prepared the samples for analysis. KZ assisted with the animal infection. TCW, HLW, HF, NF, CYW, YWG, and GH participated in sample preparation. TX assisted with the analysis of the microarray data. CQ, STY, and XZX coordinated the research efforts and edited the manuscript. XZX is the corresponding author and STY is the co-corresponding author. All authors have read and approved the final manuscript.

## Supplementary Material

Additional file 1 **Figure S1.** Target prediction of differentially expressed miRNAs upon RABV infection. Three databases TargetScan, MicroCosm Targets, and miRanda, were used to infer the targets of differentially expressed miRNAs. Click here for file

Additional file 2 **Table S1.** Significantly enriched GO terms among targets of differentially expressed miRNAs. Click here for file

Additional file 3 **Table S2.** Significantly enriched KEGG pathways of differential expressed miRNAs in mouse brain upon RABV infection. Click here for file
