# Supplementary figures and images for: Infection with street strain rabies virus induces modulation of the microRNA profile of the mouse brain
Source: Virol J. 2012 Aug 11;9:159. doi: 10.1186/1743-422X-9-159 (PMC3549733; doi:10.1186/1743-422X-9-159)

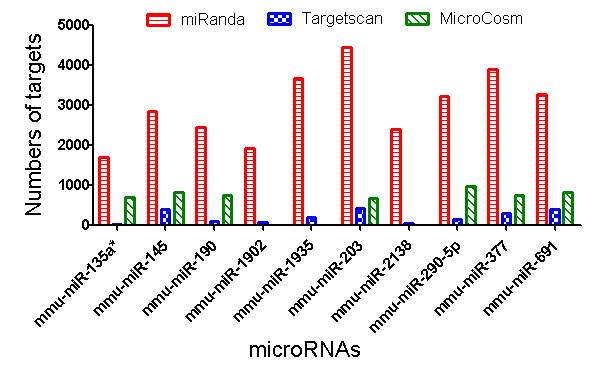

Supplement: Additional file 1 — Figure S1. Target prediction of differentially expressed miRNAs upon RABV infection. Three databases TargetScan, MicroCosm Targets, and miRanda, were used to infer the targets of differentially expressed miRNAs. [file 1743-422X-9-159-S1.tiff]
